# Experimental Investigations on Dry Sliding Wear Behavior of Kevlar and Natural Fiber-Reinforced Hybrid Composites through an RSM–GRA Hybrid Approach

**DOI:** 10.3390/ma15030749

**Published:** 2022-01-19

**Authors:** Banu Murali, Bindu Madhavan Vijaya Ramnath, Devaraj Rajamani, Emad Abouel Nasr, Antonello Astarita, Hussein Mohamed

**Affiliations:** 1 Department of Mechanical Engineering, Vel Tech Rangarajan Dr. Sagunthala R&D Institute of Science and Technology, Chennai 600062, India; rajamanitamil1991@gmail.com; 2 Department of Mechanical Engineering, Sri Sairam Engineering College, Chennai 600044, India; vijayaramnath.mech@sairam.edu.in; 3 Department of Industrial Engineering, College of Engineering, King Saud University, Riyadh 11421, Saudi Arabia; eabdelghany@ksu.edu.sa; 4 Department of Chemical, Materials and Industrial Production Engineering, University of Naples Federico II, 80138 Naples, Italy; antonello.astarita@unina.it; 5 Department of Mechanical Engineering, Faculty of Engineering, Helwan University, Cairo 11732, Egypt; hussein@h-eng.helwan.edu.eg; 6 Mechanical Engineering Department, Faculty of Engineering, Ahram Canadian University, Giza 8655, Egypt

**Keywords:** hybrid polymer composites, dry sliding wear behavior, D-optimal design, grey relational analysis, surface morphology, optimization

## Abstract

The present work aimed to investigate the dry sliding wear behaviors of hybrid polymer matrix composites made up of Kevlar, bamboo, palm, and Aloe vera as reinforcement materials of varying stacking sequences, along with epoxy as the matrix material. Three combinations of composite laminates with different stacking sequences such as AB, BC, and CA were fabricated by a vacuum-assisted compression molding process. The influence of composite laminates fabricated through various stacking sequences and dry sliding wear test variables such as load, sliding distance, and sliding velocity on the specific wear rate and co-efficient of friction were investigated. Experiments were designed and statistical validation was performed through response surface methodology-based D-optimal design and analysis of variance. The optimization was performed using grey relational analysis (GRA) to identify the optimal parameters to enhance the wear resistance of hybrid polymer composites under dry sliding conditions. The optimal parameters, such as composite combinations of CA, a load of 5 N, a sliding velocity of 3 m/s, and a sliding distance of 1500 m, were obtained. Furthermore, the morphologies of worn-out surfaces were investigated using SEM analysis.

## 1. Introduction

Bio-resources such as plant, bast, and core are widely used as natural fibers in the present manufacturing scenario for the development of novel structural materials for various high-end applications such as in the automobile, aerospace, marine industries and bioelectronics. In general, biologically derived fibers are significantly used due to their easily degradable quality and their rejuvenation of bio-active chemicals from natural processes [1]. Moreover, naturally derived fibers are more efficient than other commercial chemical fibers due to their lower density, non-abrasive manner, enhanced acoustic properties, adequate explicit modulus and strength, cost effectiveness, easy biodegradability, and efficient re-cycle capability, which improve the characteristics of natural fibers [2,3,4,5,6].

Despite their potential benefits, the inferior mechanical strength, moisture absorption, and chemical affinity of natural fibers mean they are cumbersome in practical implications [7,8,9]; hence, the hybridization of synthetic fibers with biological fibers is required to improve the mechanical properties and durability of composites [10]. Synthetic and natural fibers can be merged into an equal matrix to yield hybrid composites that take complete advantage of the finest assets of the ingredients, and in this way, an optimal cost-effective composite can be developed.

In recent years, global researchers have increased their attention on using castoff synthetic fiber resources, including glass, carbon, aramid, and other types of polymer fibers, due to their incline in the ecological risk of pollution and removal of waste at the end of life. Surplus synthetic fibers are richly available from diversified sources, including polymer fibers, glass, fabric clippings in composites, and textile industry waste [11]. Hybrid polymer matrix composites (HPMCs) are extensively utilized in delicate body shells, ballistic helmets, and several other ballistic applications [12,13,14]. The mechanical, dynamic, and tribological properties of hybridized polymer matrix composites can be enhanced through several matrix and reinforcement materials, fabrication techniques, and additions of various filler materials [15,16] to increase the use of HPMCs in a wide range of technological applications, especially in the high loading and relation motion of assembling parts. Due to the relative motion under various loading conditions, it is desirable to improve the tribological performance of composites, so that they can be effectively used for practical applications. Recently, several researchers have studied the influence of various fabrication techniques, fiber and matrix materials, and inclusion of filler materials on tribological properties such as the wear resistance of HPMCs.

Sneha Latha et al. [17] studied the effect of various woven fiber mat layering sequences in bamboo/glass fiber-reinforced hybrid composites, showing highly influenced wear behavior. An increase in the amount of glass fiber in the hybrid composites showed better mechanical properties. Similarly, increasing the amount of bamboo fiber in the hybrid composites resulted in better wear resistance, which was due to the strong interfacial bond strength between the fibers and fiber matrix. A proper combination of glass and bamboo fibers can produce hybrid composites with better mechanical properties and tribological behavior. Thus, the achievement of hybrid composites with better properties upon addition of bamboo fiber generates scope for cost reduction without compromising the quality of composites. Nevin Gamze et al. [18] investigated the performance of short fibrous composites on various parameters such as fiber alignment, length, and loading. In the fabrication process, long fibers were exposed to high-temperature shear action due to the interaction with thermoplastic resin, and this caused the fiber to twist, followed by a reduction in length. The influence of fiber matrix interfacial adhesion and test operating variables such as applied load, sliding distance, and velocity plays a major role in calculating the strength and tribo-performance of composites. Hiral et al. [19] listed the various disadvantages of using synthetic fibers for reinforcement, such as the biodegradability of fibers, high processing cost, recyclability, and health hazard. These issues can be tackled by using natural fibers as an alternative to synthetic fibers, providing an advantage in terms of the strength-to-weight ratio, ease of availability, and biodegradability. From the test results, they concluded that the hybrid basalt banana epoxy composite is a good substitute over various conventional materials, and that hybridization reduces the water absorption rate. Shuhimi et al. [20] experimentally investigated the tribological characteristics of oil palm and kenaf fiber with epoxy resin as the medium, and both composites were compared. A pin-on-disk test was administered to work out the wear behavior, and a dry sliding test was performed using a pin-on-disk tribometer. Test parameters such as weight percentage of fiber, load, sliding speed, and temperature ranges were taken as the operational parameters. The results showed that an increase in temperature resulted in a decreased coefficient of friction and an increased wear rate for both composites. The wear rate for both fibers exhibited better performance by increasing the fiber content.

From the literature, it can be inferred that several researchers have studied the tribological properties of HPMCs through experimental studies and the adoption of several statistical approaches. However, investigations pertaining to the tribological properties of a combined Kevlar, bamboo, Aloe vera, and palm-reinforced epoxy matrix composite with different stacking sequences have not been conducted by any researchers. Therefore, the present work aimed to investigate the tribological properties, such as the specific wear rate (*SWR*) and the coefficient of friction (*COF*), of various stacking sequence, such as aloe vera and bamboo (AB), bamboo and palm (BC), and palm and aloe vera (CA), with alternate layers of Kevlar-reinforced polymer matrix composites. The composite laminates were fabricated through a custom-developed vacuum-assisted compression-molding process and the wear characteristics of different stacking-sequenced HPMCs were investigated through a dry sliding wear testing apparatus with varying loading conditions, such as applied load, sliding velocity, and sliding speed. The wear test experiments were designed and executed through a categoric factored response surface methodology-based D-optimal design. The statistical validation of the proposed experimental approach was evaluated through a multiple-variable analysis of variance (ANOVA) and normal probability residual plots. The influence of various dry sliding parameters and composite combinations on the tribological properties were studied using three-dimensional response surface plots. The optimal wear testing parameters and the composite combinations were obtained through adopting the grey relational analysis approach. Furthermore, the surface modifications of dry sliding worn out surfaces were investigated through scanning electron microscopic analysis.

## 2. Proposed Methodology

### 2.1. RSM-Based D-Optimal Design

A well-organized experimental strategy can significantly influence the consequences and outcomes of experimental results. Among the several designs of experiments approaches, response surface methodology is one of the most widely used approaches for designing and executing experiments with minimal runs for novel engineering applications. In the present study, a response surface methodology (RSM)-based D-optimal design methodology was utilized to plan experiments with minimal runs [21]. The scheme of the present study utilized three numerical factors associated with wear testing and one categoric factor, i.e., different composite combinations. According to the experimental strategy, a three-level and four-factor experiment of design with a total of 28 experiments were designed. The selected parameters and their associated levels are listed in Table 1.

### 2.2. Grey Relational Analysis (GRA)

GRA is a statistical optimization methodology that can be conceived for solving complex real-world problems with inferior, inadequate, and indeterminate data sets [22]. GRA is utilized to resolve the multifaceted correlation that exists between the multiple response features of a process. The grey theory consists of two data sets, in which unidentified and well-known data are defined as “black” and “white” systems, respectively, whereas partially defined data are defined as “grey” systems.

The present investigation concentrated on the dry sliding wear behaviors, such as *SWR* and *COF*, which were initially standardized from zero to one. In this case, the selected response features should be minimized to improve the wear resistance of fabricated hybrid composites. Therefore, the characteristics of the optimization problem were proposed as “the lower, the better”. The responses of the present study were minimized by the normalization process, as mentioned by the following equation:

(1) $$S_{i}^{*}\left(n\right)=\frac{\max S_{i}\left(n\right)-S_{i}\left(n\right)}{\max S_{i}\left(n\right)-\min S_{i}\left(n\right)}$$

To achieve a considerable objective, the normalized data were standardized using the following relation:

(2) $$S_{i}^{*}\left(n\right)=1-\frac{\left|S_{i}^{o}\left(n\right)-S^{o}\right|}{\max S_{i}^{o}\left(n\right)-S^{o}}$$

The grey relational coefficient (GRC) is defined as the relationship factor between the experimental standardized data and the actual data. The GRC can be calculated using the following expression:

(3) $$\xi_{i}\left(n\right)=\frac{\Delta_{\min}+\xi \Delta_{\max}}{\Delta_{oi}\left(n\right)+\xi \Delta_{\max}}$$

where the deviation of the reference sequence order is indicated as $\Delta_{oi}$ and the comparability sequence is indicated by Δmax.

(4) $$\begin{array}{l} \Delta_{oi}=\left\|S_{o}^{*}\left(n\right)-S_{i}^{*}\left(n\right)\right\|\\ \Delta_{\min}=\underset{\forall j∍i}{\min}\;\underset{\forall k}{\min}\left\|S_{o}^{*}\left(n\right)-S_{j}^{*}\left(n\right)\right\|\\ \Delta_{\min}=\underset{\forall j∍i}{\max}\;\underset{\forall k}{\max}\left\|S_{o}^{*}\left(n\right)-S_{j}^{*}\left(n\right)\right\| \end{array}$$

where the reference order is $S_{o}^{*}\left(n\right)$ and $S_{i}^{*}\left(n\right)$ indicates the comparability order. The identification factor is defined by ξ. The grey relational grade (GRG) is calculated by:

(5) $$\gamma_{i}=\frac{1}{m}\sum_{n=1}^{m}\xi_{i}\left(n\right)$$

where the number of total experimental trials is indicated as *m*, and the GRG is indicated as $\gamma_{i}$, which lies between zero to one.

## 3. Experimental Details

### 3.1. Materials

Aramid is an acronym for aromatic polyamide (poly-p-phenylene terephthalamide), often known as Kevlar, which is the most widely used synthetic fiber in the fabrication of components for aerospace and structural applications. Many compostable and biodegradable fibers produced from plants have also been used as reinforcement components in polymer for the production of sophisticated composites by researchers. Natural fibers such as aloe vera, bamboo, and palmyra palm, and Kevlar fiber with densities of 1.4, 0.9, 1.3, and 1.43 g/cm^3^ were employed in a predetermined stacking sequence to fabricate the hybrid composites in this study. The matrix material was formed of epoxies (LY556) and hardeners (HY951) with densities of 1.15–1.20 g/cm^3^ and 0.97–0.99 g/cm^3^, respectively. Kevlar fibers were used as the outer skin of the fabricated composite to enhance the surface quality and strength. The weight ratio of the mixing epoxy and hardener was 10:1 to provide adequate interfacial bonding between fibers.

### 3.2. Fabrication of Composites

**Figure 1:**
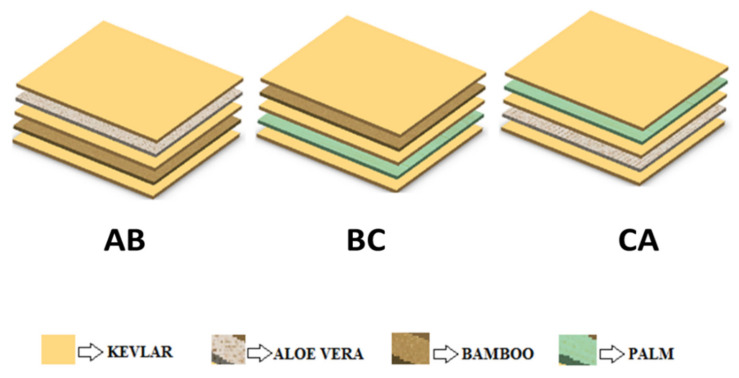
The configuration of composites fabricated with varying stacking sequences.

**Figure 2:**
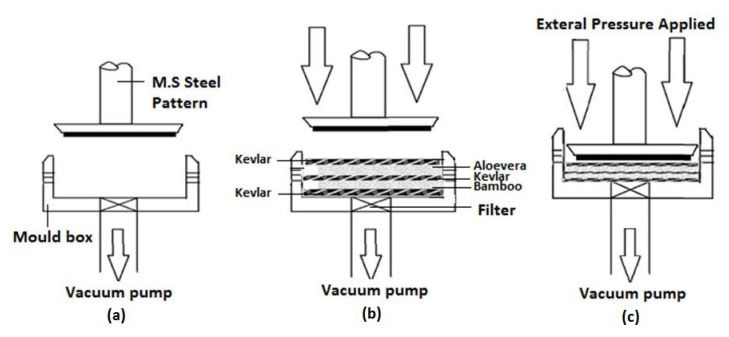
Schematic of vacuum-assisted compression molding for the fabrication of hybrid composites: (**a**) Mold and pattern; (**b**) arrangements of prepregs in the mold; (**c**) compression of prepregs with external pressure.

The composite laminates for the present investigation were fabricated through a vacuum-assisted compression molding process due to its prominent benefits over other conventional processing techniques. The composite specimens were fabricated with five layers, in which the outer and middle layers were made up of Kevlar fiber and the other layers, such as each second and fourth layer, were made up of Aloe vera, bamboo, and palm fibers. The stacking sequence of the fibers for different composite laminates are shown in Figure 1, and the same is presented in Table 2. For each hybrid composite, the ratio of fibers to the polymer matrix was taken as 60:40. In this proposed vacuum-assisted compression molding process, the downsides of different techniques were overwhelmed by making a vacuum inside the shape, with the goal that an air trap could not be framed inside the composite. The phases of fabricating composite laminates through the proposed approach are presented in Figure 2a–c. In the first stage of fabrication, a shield (mold box), along with a vacuum pump and an MS steel pattern, was prepared with the prescribed shape in which the composite was fabricated. In the wake of framing the necessary blend, the shape was permitted to dry for 5 h, and afterward, the manufactured composite overlay was launched out from the mold. After the composite laminates had solidified, the unpleasant edges were ejected and cut according to the required dimensions.

### 3.3. Wear Test and Measurement

According to the RSM-based D-optimal experimental strategy, the fabricated composite laminates (AB, BC, and CA) were machined to obtain test samples for wear testing with appropriate dimensions (6 mm diameter and 24 mm length). The experiments were performed using a pin-on-disk setup (Model: Ducom-TR-20LE) to obtain the wear characteristics. To obtain the wear characteristics, the counterface disk (100 mm diameter) was made up of a highly hardened die steel with a hardness value of 62 HRC. As per the experimental strategy, a total of 28 experiments were performed to attain the *SWR* and *COF* values. The *SWR* of the test specimens was assessed through the following relation:

(6) SWRmm3Nm=Loss of volumeSliding distance × Load

The coefficient of friction for each experimental trial was directly obtained from the wear testing apparatus. The response values were assessed thrice for each experiment to avoid measuring errors, and their corresponding values are presented in Table 3.

## 4. Results and Discussion

### 4.1. Statistical Analysis and Development of an Empirical Model

**Figure 3:**
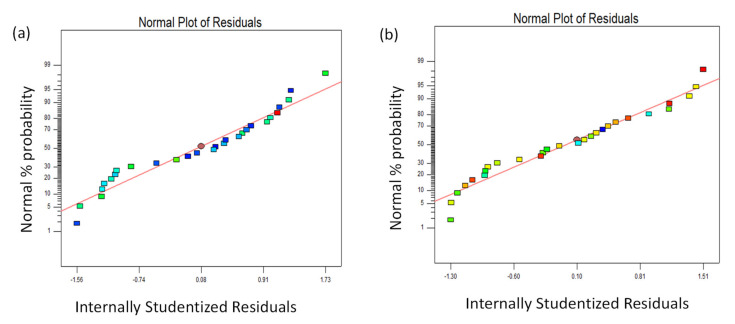
Normal probability plot of residuals for each response: (**a**) *SWR*; (**b**) *COF*.

The statistical ANOVA results for *SWR* are presented in Table 4. From the ANOVA, the *F*-value of 54.04 suggests that the model is passable for effectively modeling *SWR*. Moreover, the model’s “Prob > *F*” values for all of the parameters are <0.0005. The coefficient of determination (*R*^2^) for the coefficient of friction is 98.92% and the adjusted coefficient of determination (Adj. *R*^2^) is 97.09%, which indicates that the experimental data are sensible and the relationship produced is agreeable. Moreover, model validation analysis was performed using a normal probability plot. The response of the normal plot for the coefficient of friction is shown in Figure 3a and seems to be normally distributed, and it is similar to a straight line, which denotes settled significance between the models. Furthermore, it can be observed that the residuals set against with predicted plots are normally distributed.

The statistical ANOVA results for *COF* are presented in Table 5. From the ANOVA, the *F*-value of 9.9335 suggests that the model is passable for effectively modeling the coefficient of friction. Moreover, the model “Prob > *F*” values for all of the parameters are <0.0005. The coefficient of determination (*R*^2^) for the coefficient of friction is 94.40% and the adjusted coefficient of determination (Adj. *R*^2^) is 84.90%, which indicates that the experimental data are sensible and the relationship produced is agreeable. Moreover, model validation analysis was performed using a normal probability plot. The response of the normal plot for the coefficient of friction is shown in Figure 3b and seems to be normally distributed, and it is similar to a straight line, which denotes settled significance between the models. Furthermore, the residuals set against with predicted plots are normally distributed. Hence, the developed mathematical models can be effectively utilized for further investigations, and there is no motive to insure any desecration of independence [23].

### 4.2. Influence of the Process Parameters on the Wear Characteristics

#### Influence on *SWR*

**Figure 4:**
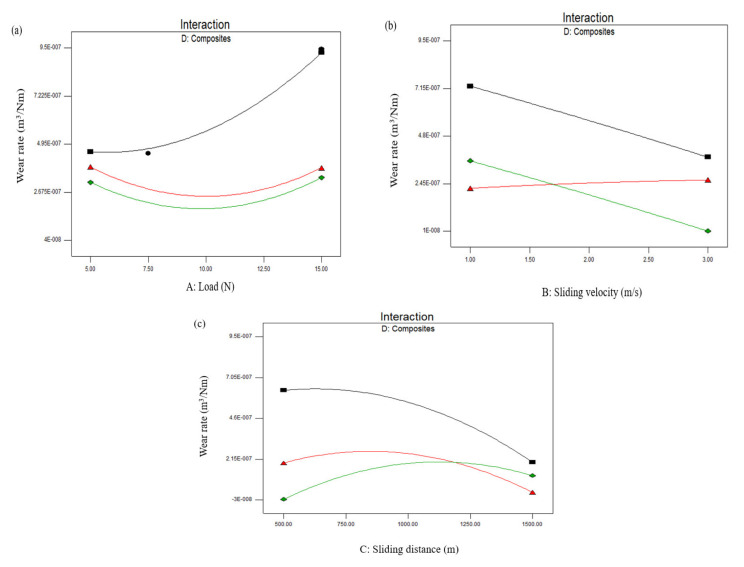
Two-dimensional interaction plots for the specific wear rate: (**a**) Wear rate vs. load; (**b**) wear rate vs. sliding velocity; (**c**) wear rate vs. sliding distance. (Composite combinations AB—Black; AC—Green; and BC—Red).

From the statistical analysis, it was found that the selected parameters have a significant influence on *SWR*. The significant variations of *SWR* with respect to the loading conditions of the three different composites are presented in the interaction plots (Figure 4a–c). The influence of the load conditions and the different composite laminates on *SWR* are presented in Figure 4a. From the plot, it can be inferred that the wear rate of the composite laminates significantly increased with the increase of the applied load from 5 to 15 N. It can be seen from the plot that the *SWR* of composite D1 (AB) seems to be prominent, followed by D2 (BC), whereas an improved wear resistance was found for composite D3 (CA). These can be attributed to the fact that the rate of wear increases in a non-linear fashion from standard to severe, and this growing trend was observed under different load conditions. Similar trends were observed in the adhesive wear properties of an aluminum hybrid metal matrix composites at a higher temperature, where their rate of wear increased in a non-linear fashion under high-velocity conditions [24].

The impact of sliding velocity and the composite combinations on *SWR* are depicted in Figure 4b. From the interaction plot, it is evident that the *SWR* of composite combinations D1 (AB) and D3 (AC) slightly decreased with the increase in sliding velocity, whereas the *SWR* of composite D2 (BC) was found to be constant throughout the sliding velocity from 1 to 3 m/s. The influence of sliding distance and different composite combinations on *SWR* are shown in the interaction plot in Figure 4c. From the plot, it can be observed that composite D3 (CA) exhibited an improved wear resistance with minimized *SWR* at a lower sliding speed. This could be due to the fact that hard-reinforced particles protruded at the specimen surface and bumped into the counterface at a lower sliding distance, and greater removal of the material was observed. Moreover, it can be seen that the wear rate slightly decreased with the increase in sliding distance for composite combinations D1 (AB) and D2 (BC).

Hence, it is desirable to decrease the sliding distance to improve the quality of cut through decreasing the *SWR*. The percentage of all three composites (AB, BC, and CA) led to a sensible decline in the rate of wear rate, as displayed in Figure 4a–c. Similar to our present findings, an enhanced hardness increment on the polyether ether ketone matrix generated enhanced the resistance in the wear and seizure materials [21].

**Figure 5:**
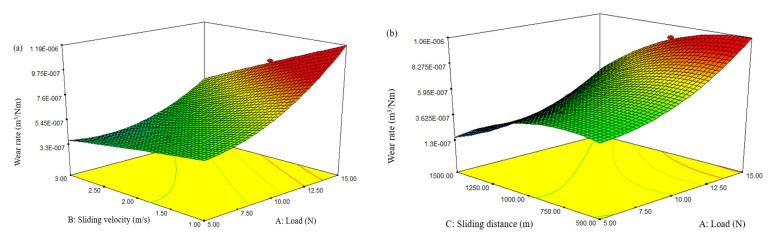
3D response surface plots for the wear rate: (**a**) Load vs. sliding velocity; (**b**) load vs. sliding distance.

The response surface plots of composite D3 (CA) are displayed in Figure 5a,b, displaying the interface effect of diverse processing conditions on *SWR*. The influence of sliding velocity and load on *SWR* are presented in Figure 5a. It can be observed from the plot that the *SWR* of the hybrid composite significantly increased with the rise of both variables. However, the minimized *SWR* was experiential for combinations of a lower loading condition (5 N) and a higher sliding velocity (3 m/s).

The impact of sliding distance and load on the *SWR* of composite D3 (CA) are described in Figure 5b. The plot shows that the *SWR* of the hybrid composites drastically increased with the upsurge of load and sliding distance. This could be attributed to the oxidation of composites during high loading conditions. During higher loading conditions, the frictional force between the disk and test specimens increase, which leads to an augmented *SWR* [21].

#### Influence on *COF*

**Figure 6:**
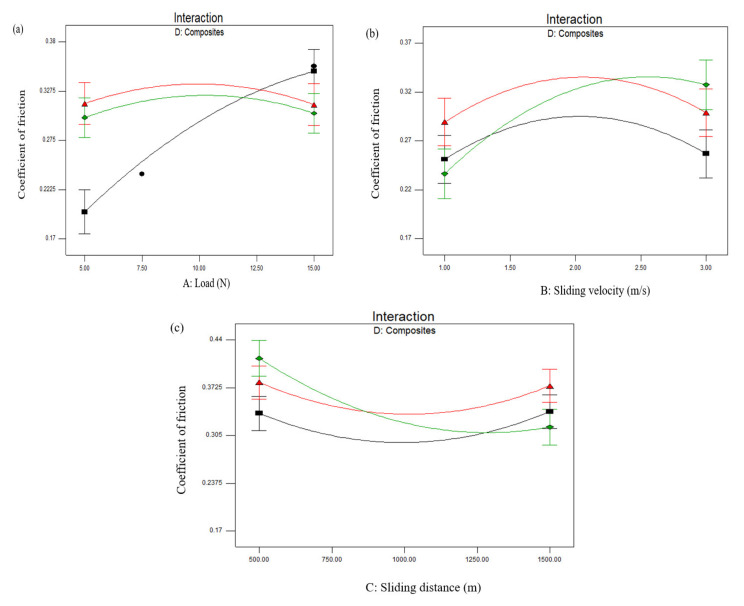
Two-dimensional interaction plots for *COF*: (**a**) Load vs. *COF*; (**b**) sliding velocity vs. *COF*; (**c**) sliding distance vs. *COF*. (Composite combinations AB—Black; AC—Green; and BC—Red).

The statistical validation results indicate that the selected wear and composite variables have a substantial impact on *COF*. Figure 6a–c indicates the interaction influence of the process parameters and various combinations of fabricated laminates on *COF*. The influence of the applied load and the composite combinations on *COF* are depicted in Figure 6a. The interaction plot indicates that *COF* increased with the increase in load for the composite D1 (AB), whereas there was no obvious change in the *COF* for the other two composite combinations. As the load increased, the area of contact between the pin and disk increased and, hence, *COF* also increased. The minimal *COF* was found at lower loading condition (5 N) for the D1 (AB) composite.

The influence of sliding velocity and the composite combinations on *COF* are presented in Figure 6b. The plot clearly indicates that *COF* slightly increased with the increase in sliding velocity for all three combinations of composites. However, a minimal *COF* was observed for composite D3 (CA). This is due to the fact that increasing the friction coefficient with sliding velocity leads to increased adhesion of the counterface material (pin) on the disk [21]. The variation of *COF* with respect to the sliding distance for different composites is presented in Figure 6c. The interaction plot indicates that there was no obvious change in *COF* for the composites. However, composites D1 (AB) and D3 (CA) exhibited lower *COF*s compared to composite D2 (BC). This is mainly because the matrix layer of D1 (AB) and D3 (CA) contacts the load carried by these fiber matrices, and also, *COF* declined compared to D2 (BC). The effectiveness of *COF* rests on the amount of load carried by the matrix/matrix, fiber/matrix, and fiber/fiber contacts and their respective *COF*s.

**Figure 7:**
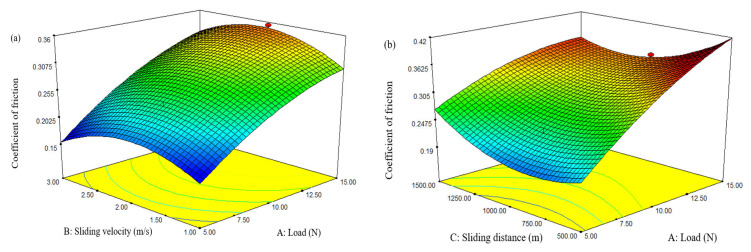
3D response surface plots for *COF*: (**a**) Load vs. sliding velocity; (**b**) load vs. sliding distance.

From the interaction plots, it was found that composite D3 (CA) exhibited lower *COF*, which is similar to the wear rate. Therefore, composite D3 (CA) was selected for further investigation. The 3D response surface plots shown in Figure 7a,b indicate the influence of the dry sliding parameters on the *COF* of composite combination D3 (CA). The consequence of sliding velocity and load on *COF* is presented in Figure 7a. It can be observed from the plot that *COF* linearly increased with respect to the applied load, whereas there was no significant variation in the sliding velocity. However, a lower *COF* was observed at lower values of load (5 N) and sliding velocity (1 m/s). The increase in frictional coefficient with the increase in load rate was mainly due to the upsurge of adhesion strength. Generally, in many composite pairs, *COF* is low at the lower loads and a transition arises to a higher rate as the normal load increases [25].

Similarly, the consequences of sliding distance and load on *COF* are displayed in Figure 7b, which indicates that the increase in load rate and sliding distance from lower to higher values increases *COF* to maximum values. The augmented *COF* is experienced as a result of frictional heat, which is produced at countersurfaces at a higher loading condition. A simultaneous improvement in the applied load with sliding distance will improve the frictional force at the contact surface, and hence, *COF* will increase. However, a lower *COF* was found at a lower load (5 N) and sliding distance (500 m). From the investigations, it is observed that the *COF* of hybrid composites can be minimized by reducing the applied load, sliding velocity, and sliding distance.

### 4.3. Microstructural Analysis of Worn-Out Surfaces

**Figure 8:**
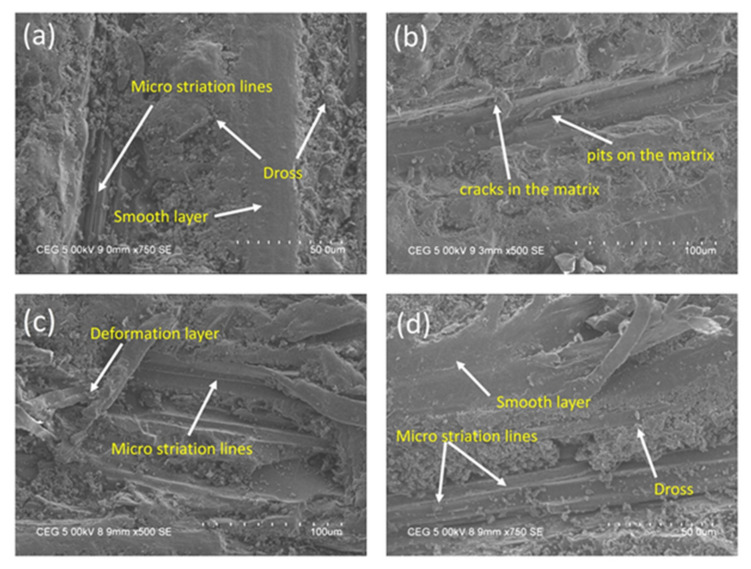
The SEM analysis of worn-out specimens at different wear testing conditions: (**a**,**b**) Load of 5 N, sliding velocity of 1 m/s, and sliding distance of 500 m; (**c**,**d**) load of 15 N, sliding velocity of 3 m/s, and sliding distance of 1500 m.

Microstructure screening of worn-out surfaces can deliver a better vison of the quality of fabricated hybrid composites with respect to different compositions and working conditions. The microstructures of worn-out surfaces were investigated using a scanning electron microscope (model: Sigma 300, Carl Zeiss, Oberkochen, Germany) with an accelerating peak power of 9–15 keV. The scanning electron microscopic (SEM) images of three different bio composites (AB, BC, and CA) with various processing conditions are displayed in Figure 8a,d. From the experimental investigations, it was found that composite CA delivered a significant wear resistance characteristic, and it was screened for SEM analysis under various processing conditions. Figure 8a,b indicate a worn-out surface of composite CA at a testing condition of a 5 N load, a 1 m/s sliding velocity, and a 500 m sliding distance. The micrographs show a minimal plow up track, micro-striation pattern, and smooth surface with reduced voids and pores, which indicates the eventual bonding between the reinforced and matrix materials. Balan et al. [26] found that the SEM images of epoxy-based hybrid composites demonstrated stable bonding and compatibility between the matrix and reinforced materials, with small fissures originating at the plastic particle interface and the matrix. Figure 8c,d show the SEM micrographs of wear surfaces of the composite combination CA at an operating condition of a 15 N load, a 3 m/s sliding velocity, and a 1500 m sliding distance. It is evident from the micrographs that long pits and grooves, delamination, and fiber breakout with deep striation patterns occur at higher loading conditions. As the applied load increases, the wear characteristics of the fiber composites varies from abrasion to delamination, as apparent from the surface morphologies. The initiation of cracks and delamination at the interface of the particulates lead to de-bonding and pits formation [26]. The deformation of the composite surface increases due to the higher relative motion between the pin and disk, which significantly increases the wear rate of composites under higher loading conditions.

### 4.4. Prediction of Optimal Process Variables Using GRA

The goal of this research was to improve the wear properties of cast-off hybrid composite laminates by reducing *SWR* and *COF*. With the help of Equation (1) in GRA, the response variables were first normalized based on “the smaller, the better the conditions.” Equation (2) was used to calculate the GRC values, and the grey relational grade (GRG) values were calculated by allocating equal weight to each of the responses studied in this study.

**Figure 9:**
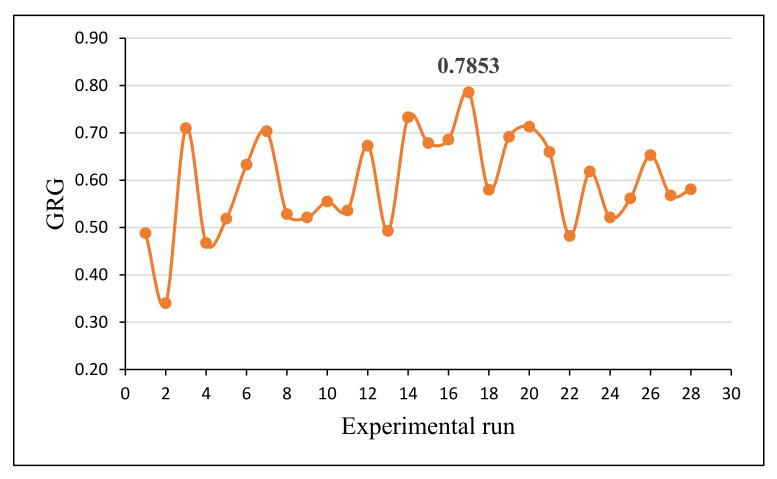
Graphical representation of the obtained grey relational grade for each experimental run.

The obtained GRG of *SWR* and *COF* for different experimental runs are mentioned in Table 6. The fraction between the comparability and sequence of reference was defined using GRG. The optimal processing variable combination was obtained through taking the mean of the GRG values. The maximum grey relational grade of 0.7853 was obtained in the 17th run (Rank I) and it is significant with other grey relational grades. The corresponding process parameters, such as of composite combination of D1 (CA), i.e., a load of 5 N, a sliding velocity of 3 m/s, and a sliding distance of 1500 m, were considered optimal parameters of the composites for obtaining improved wear properties. A graphical representation of the obtained GRG for various experimental runs is presented in Figure 9.

## 5. Conclusions

The present investigation aimed to envisage the dry sliding wear characteristics of fabricated hybrid polymer matrix composites of three different compositions: AB—aloe vera and bamboo; BC—bamboo and palm; and CA—palm and aloe vera. The results of the experimental, modeling, and optimization studies are as follows:

- The dry sliding wear test experiments were designed and executed through a D-optimal design approach. The ANOVA results displayed that the proposed quadratic mathematical models are efficient at 95% conformance levels in the prediction of wear behaviors of fabricated composites with a coefficient of determination of 98.92% for *SWR* and 97.09% for *COF*.
- From the ANOVA and interaction results, composite D3 (CA) showed improved wear resistance due to improved interfacial bonding and stacking sequence, even at higher loads and sliding conditions. Moreover, the sliding distance was found to be more significant parameters for a specific wear rate, whereas the applied load and sliding distance were found to be significant for *COF*.
- Based on the RSM–GRA hybrid approach, the optimal parameter combinations for improved wear properties were obtained and are as follows: Composite combinations of D3 (CA), a load of 5 N, a sliding velocity of 3 m/s, and a sliding distance of 1500 m with a maximum GRG of 0.7853.
- SEM micrographs revealed the micro-striation patterns and fiber delamination of worn-out surfaces under various testing conditions.
- It is proposed that the hybrid approach of RSM–GRA can be effectively utilized for the modeling and optimization of the wear properties of hybrid polymer matrix composites with minimal computational effort within the selected limits of processing conditions.
- Future perspectives of the present research may enlighten the investigation of thermal and dynamic properties of the composites with different orientations and stacking methods.

## Figures and Tables

**Table 1:** Process variables with their levels used for dry sliding wear tests.

No	Process Variables	Unit	Levels		
			−1	0	1
1	Applied load	N	5	10	15
2	Sliding velocity	m/s	1	2	3
3	Sliding distance	m	500	1000	1500
4	Composite combination	Categorical	AB	BC	CA

**Table 2:** Proposed stacking sequence of composite laminates.

D1 (AB)	D2 (BC)	D3 (CA)
Kevlar	Kevlar	Kevlar
Aloe vera	Bamboo	Palm
Kevlar	Kevlar	Kevlar
Bamboo	Palm	Aloe vera
Kevlar	Kevlar	Kevlar

**Table 3:** Experimentally measured *SWR* and *COF* for the composite specimens.

Exp No.	Applied Load (N)	Sliding Velocity (m/s)	Sliding Distance (m)	Sequence of Composite Laminates	*SWR* (mm^3^/N-m)	*COF*
1	10	3	500	AB	3.82 × 10^−7^	0.323
2	15	2	1000	AB	9.43 × 10^−7^	0.354
3	15	1	1500	BC	1.81 × 10^−7^	0.266
4	15	1	500	CA	4.50 × 10^−7^	0.318
5	15	3	500	BC	2.72 × 10^−7^	0.357
6	15	2	1500	CA	2.10 × 10^−7^	0.268
7	15	3	1000	CA	7.43 × 10^−8^	0.316
8	10	1	1500	AB	3.73 × 10^−7^	0.285
9	10	1	1500	AB	3.14 × 10^−7^	0.319
10	15	3	1500	AB	2.56 × 10^−7^	0.322
11	5	3	500	BC	3.24 × 10^−7^	0.297
12	10	1.5	1250	BC	1.04 × 10^−7^	0.313
13	10	3	500	AB	4.61 × 10^−7^	0.287
14	5	1	500	AB	5.74 × 10^−7^	0.17
15	5	1	1500	BC	6.50 × 10^−8^	0.37
16	5	1	1500	BC	7.71 × 10^−8^	0.335
17	5	3	1500	CA	1.06 × 10^−7^	0.226
18	5	3	1500	CA	2.25 × 10^−7^	0.308
19	10	3	1500	BC	7.16 × 10^−8^	0.336
20	5	2	500	CA	4.25 × 10^−8^	0.36
21	10	2.5	1250	CA	1.08 × 10^−7^	0.325
22	12.5	2	750	BC	3.31 × 10^−7^	0.365
23	5	1	1000	CA	3.96 × 10^−7^	0.22
24	5	3	500	BC	3.73 × 10^−7^	0.29
25	7.5	2	1000	AB	4.51 × 10^−7^	0.239
26	15	1	1500	BC	1.45 × 10^−7^	0.293
27	10	1.5	750	CA	2.16 × 10^−7^	0.33
28	10	1	500	BC	2.01 × 10^−7^	0.327

**Table 4:** Analysis of variance for *SWR*.

Source	Sum of Squares	DOF	Mean Square	*F*-Value	Prob > *F*	Remarks
Model	1.03 × 10^−12^	17	6.09 × 10^−14^	54.04	<0.0001	Significant
A-Load	3.11 × 10^−14^	1	3.11 × 10^−14^	27.61	0.0004	
B-Sliding velocity	9.69 × 10^−14^	1	9.69 × 10^−14^	85.99	<0.0001	
C-Sliding distance	1.43 × 10^−13^	1	1.43 × 10^−13^	127.6	<0.0001	
D-Composites	4.51 × 10^−13^	2	2.25 × 10^−13^	200.13	<0.0001	
AB	9.02 × 10^−14^	1	9.02 × 10^−14^	80.05	<0.0001	
AC	3.73 × 10^−14^	1	3.73 × 10^−14^	33.08	0.0002	
AD	1.36 × 10^−13^	2	6.81 × 10^−14^	60.41	<0.0001	
BC	9.73 × 10^−15^	1	9.73 × 10^−15^	8.63	0.0148	
BD	1.37 × 10^−13^	2	6.86 × 10^−14^	60.83	<0.0001	
CD	1.65 × 10^−13^	2	8.29 × 10^−14^	73.55	<0.0001	
A^2^	9.51 × 10^−14^	1	9.51 × 10^−14^	84.37	<0.0001	
C^2^	5.32 × 10^−14^	1	5.32 × 10^−14^	47.19	<0.0001	
Residual	1.12 × 10^−14^	10	1.12 × 10^−15^			Not significant
Lack of Fit	5 × 10^−15^	5	1 × 10^−15^	0.79	0.5951	
Pure Error	6.27 × 10^−15^	5	1.25 × 10^−15^			
Cor. Total	1.04 × 10^−12^	27				
	*R*^2^ = 98.92%		Adj. *R*^2^ = 97.09%			

**Table 5:** Analysis of variance for the coefficient of friction (ANOVA).

Source	Sum of Squares	DOF	Mean Square	*F*-Value	Prob > *F*	Remarks
Model	0.057	17	0.003	9.933	0.0004	Significant
A-Load	0.002	1	0.002	8.082	0.0175	
B-Sliding velocity	0.001	1	0.001	4.728	0.0458	
C-Sliding distance	0.000	1	0.000	1.957	0.0192	
D-Composites	0.009	2	0.004	13.884	0.0013	
AB	0.000	1	0.000	1.515	0.0246	
AC	0.009	1	0.009	26.680	0.0004	
AD	0.014	2	0.007	20.826	0.0003	
BC	0.000	1	0.000	1.577	0.0237	
BD	0.004	2	0.002	6.284	0.0171	
CD	0.005	2	0.002	8.414	0.0072	
A^2^	0.002	1	0.002	6.931	0.0250	
B^2^	0.004	1	0.004	14.124	0.0037	
C^2^	0.004	1	0.004	13.550	0.0042	
Residual	0.003	10	0.000			
Lack of Fit	0.001	5	0.000	0.520	0.7547	Not significant
Pure Error	0.002	5	0.000			
Cor.Total	0.060	27				
	*R*^2^ = 94.4%		Adj. *R*^2^ = 84.9%			

**Table 6:** Calculation of grey relational coefficients and grey grades.

Run	Grey Relational Coefficient		Grey Relational Grade	Rank
	*SWR*	*COF*		
1	0.5865	0.3892	0.4879	25
2	0.3334	0.3464	0.3399	28
3	0.9155	0.5039	0.7097	4
4	0.5373	0.3971	0.4672	27
5	0.6940	0.3427	0.5184	23
6	0.7662	0.4987	0.6324	12
7	1.0064	0.4004	0.7034	5
8	0.5974	0.4588	0.5281	20
9	0.6463	0.3955	0.5209	22
10	0.7182	0.3908	0.5545	18
11	0.6366	0.4343	0.5355	19
12	0.9401	0.4054	0.6728	9
13	0.5303	0.4545	0.4924	24
14	0.4656	1.0000	0.7328	2
15	1.0285	0.3277	0.6781	8
16	0.9999	0.3714	0.6857	7
17	0.9355	0.6352	0.7853	1
18	0.7448	0.4140	0.5794	15
19	1.0127	0.3700	0.6914	6
20	1.0866	0.3391	0.7129	3
21	0.9327	0.3861	0.6594	10
22	0.6307	0.3333	0.4820	26
23	0.5756	0.6610	0.6183	13
24	0.5936	0.4483	0.5210	21
25	0.5366	0.5856	0.5611	17
26	0.8630	0.4422	0.6526	11
27	0.7567	0.3786	0.5676	16
28	0.7780	0.3831	0.5805	14

## Data Availability

Data is contained within the article.

## References

[1] Sastra H.Y., Siregar J.P., Sapuan S.M., Hamdan M.M. (2007). Tensile Properties of Arenga pinnata Fiber-Reinforced Epoxy Composites. Polym. Plast. Technol. Eng..

[2] Leman Z., Sapuan S.M., Azwan M., Ahmad M.M.H.M., Maleque M.A. (2008). The Effect of Environmental Treatments on Fiber Surface Properties and Tensile Strength of Sugar Palm Fiber-Reinforced Epoxy Composites. Polym. Plast. Technol. Eng..

[3] Uyup M.K.A., Paridah M.T., Husain H., Sapuan S.M., Bakar E.S. (2009). Effect of curing time on physical and mechanical properties of phenolic-treated bamboo strips. Ind. Crops Prod..

[4] Sapuan S.M., Harimi M., Maleque M.A. (2003). Mechanical Properties of Epoxy/Coconut Shell Filler Particle Composites. Arab. J. Sci. Eng..

[5] Rashdi A.A.A., Sapuan S.M., Ahmad M.M.H.M., Khalina A. (2009). Water absorption and tensile properties of soil buried kenaf fibre reinforced unsaturated polyester composites (KFRUPC). J. Food Agric. Environ..

[6] Jawaid M., Abdul Khalil H.P.S., Abu Bakar A. (2011). Woven hybrid composites: Tensile and flexural properties of oil palm-woven jute fibres-based epoxy composites. Mater. Sci. Eng. A.

[7] Kumar N.R., Chandra R.B. (2018). Retention of Mechanical and Thermal Properties of Hydrothermal Aged Glass Fiber-Reinforced Polymer Nanocomposites. Polym. Plast. Technol. Eng..

[8] Nayak R.K., Ray B.C. (2018). Influence of seawater absorption on retention of mechanical properties of nano-TiO2 embedded glass fiber reinforced epoxy polymer matrix composites. Arch. Civ. Mech. Eng..

[9] Nayak R.K., Ray B.C. (2017). Water absorption, residual mechanical and thermal properties of hydrothermally conditioned nano-Al2O3 enhanced glass fiber reinforced polymer composites. Polym. Bull..

[10] Narayanan V., ElayaPerumal A., Alavudeen A., Thiruchitrambalam M. (2011). Mechanical and water absorption behaviour of banana/sisal reinforced hybrid composites. Mater. Des..

[11] Tufan M., Akbas S., Aslan M. (2016). Decay resistance, thermal degradation, tensile and flexural properties of sisal carbon hybrid composites. Maderas-Cienc. Tecnol..

[12] Wambua P., Vangrimde B., Lomov S., Verpoest I. (2007). The response of natural fibre composites to ballistic impact by fragment simulating projectiles. Compos. Struct..

[13] Mishra S., Mohanty A.K., Drzal L., Misra M., Parija S., Nayak S.K., Tripathy S.S. (2003). Studies on mechanical performance of biofibre/glass reinforced polyester hybrid composites. Compos. Sci. Technol..

[14] Abdul Khalil H.P.S., Hanida S., Kang C.W., Nik Fuaad N.A. (2007). Agro-hybrid Composite: The effects on mechanical and physical properties of oil palm fiber (EFB)/glass hybrid reinforced polyester composites. J. Reinf. Plast. Compos..

[15] Chaudhary V., Bajpai P.K., Maheshwari S. (2018). An Investigation on Wear and Dynamic Mechanical behaviour of Jute/Hemp/Flax Reinforced Composites and Its Hybrids for Tribological Applications. Fiber Polym..

[16] Shalwan A., Yousif B. (2013). In state of art: Mechanical and tribological behaviour of polymeric composites based on natural fibres. Mater. Des..

[17] Sneha Latha P., Venkateswara Rao M., Kiran Kumar V.V., Raghavendra G., Ojha S., Inala R. (2016). Evaluation of mechanical and tribological properties of bamboo-glass hybrid fibre reinforced polymer composite. J. Ind. Text..

[18] Karsli N.G., Aytac A., Deniz V. (2012). Effects of initial fiber length and fiber length distribution on the properties of carbon-fiber-reinforced-polypropylene composites. J. Reinf. Plast. Comp..

[19] Parikh H.H., Soni H.P., Suthar D.A., Patel D.H. (2019). Mechanical and Tribological Characterization of Hybrid Natural Fiber Reinforced Composites. Curr. Mater. Sci..

[20] Shuhimi F.F., Bin Abdollah M.F., Kalam M.A., Masjuki H.H., Mustafa A., Amiruddin H. (2016). Tribological Characteristics Comparison for Oil Palm Fibre/Epoxy and Kenaf Fibre/Epoxy Composites under Dry Sliding Conditions. Tribol. Int..

[21] Rajmohan T., Palanikumar K., Davim J.P., Arun Premnath A. (2014). Modeling and optimization in tribological parameters of polyether ether ketone matrix composites using D-optimal design. J. Thermoplast. Comp..

[22] Rajamani D., Ziout A., Balasubramanian E., Velu R., Sachin S., Mohamed H. (2018). Prediction and analysis of surface roughness in selective inhibition sintered high-density polyethylene parts: A parametric approach using response surface methodology–grey relational analysis. Adv. Mech. Eng..

[23] Balasubramanian E., Rajamani D., Arunkumar P. (2018). Modeling and prediction of optimal process parameters in wear behaviour of selective inhibition sintered high density polyethylene parts. Prog. Addit. Manufact..

[24] Aravind C., Gopala Krishnan S., Radhika N. (2019). Investigating the Adhesive Wear Properties of Aluminum Hybrid Metal Matrix Composites at Elevated Temperatures Using RSM Technique. Tribol. Ind..

[25] Nuruzzaman D.M., Chowdhury M.A. (2012). Effect of Normal Load and Sliding Velocity on Friction Coefficient of Aluminum Sliding Against Different Pin Materials. Am. J. Mater. Sci..

[26] Sakthi-Balan G., Ganesh N., Ravichandran M. (2020). Study of tribological and water intake characteristics of epoxy-based hybrid composite. Mater. Today.

